# Analysis of Intoxication, Rehospitalization, and One-year Survival of Heart Failure Patients Receiving Digoxin at Harapan Kita National Cardiovascular Center, Jakarta, Indonesia: A Cross Section-observational Study

**DOI:** 10.2174/1574886317666220520114417

**Published:** 2023-01-12

**Authors:** Nafrialdi Nafrialdi, Cindy Tiaranita, Fransiscus D. Suyatna, Bambang Budi Siswanto

**Affiliations:** 1 Department Pharmacology and Therapeutics, Faculty of Medicine, Universitas Indonesia, Jakarta, 10430, Indonesia;; 2 Department Cardiology and Vascular Medicine, Faculty of Medicine, Universitas Indonesia-Harapan Kita National Cardiovascular Center, Jakarta, Indonesia

**Keywords:** Digoxin intoxication, heart failure, risk factors, survival, rehospitalization, treatment

## Abstract

**Background:**

Despite being the oldest therapy for heart failure, the use of digoxin is still controversial due to the narrow margin of safety. In Indonesia, digoxin is still considered one of the treatments for heart failure. However, analysis of intoxication has never been reported. This study aims to analyze the occurrence of digoxin intoxication, rate of rehospitalization and one-year survival in heart failure patients under digoxin treatment.

**Methods:**

A cross section-observational study was conducted at Harapan Kita National Cardiovascular Centre from January 2017 to December 2018 on heart failure patients who received digoxin therapy and had data on serum digoxin level. Intoxication was defined as the presence of specific ECG alteration(s), at least one extra-cardiac symptom(s) and further classified as definite (serum digoxin >2 ng/mL), probable (serum digoxin 0.91-1.99 ng/mL), or possible (serum digoxin 0.5-0.9 ng/mL). Risk factors of intoxication were analyzed by Chi-square test, and one-year survival was analyzed with Kaplan Meyer method.

**Results:**

54 of 195 patients (27.69%) were classified as having intoxication, consisting of 32 (16.41%) definite, 19 (9.74%) probable, and 3 (1.54%) possible. Renal insufficiency was revealed as a significant influencing factor of digoxin intoxication with RR 2.48 (CI 1.13-5.464, *p=*0.016). Overall one-year survival of patients receiving digoxin was 259 days in the intoxication group and 307 days in the non-intoxication group. One-year rehospitalization was 11.8% in patients who received digoxin and 29.2% in those without digoxin (*p=*0.085).

**Conclusion:**

The proportion of digoxin intoxication in heart failure patients was 27.69%. Renal insufficiency was revealed as a significant influencing factor of intoxication. There was a tendency of reduced hospitalization in those who received digoxin.

## 1 INTRODUCTION

Heart failure is a global health problem that involves 38 million people all over the world [1-3]. Data from the American Heart Association reported 5.7 million people suffering from heart failure in 2009-2012 (1.5-1.9%), and it increased to 6.2 million (2.2%) in 2013-2016. It is predicted that this number will reach 8.5 by 2030 [4, 5].

Prevalence of heart failure in Europe and Asia is quite similar to that of global prevalence (1-3%), except in Taiwan and Indonesia, which reach >5% [2, 3]. Heart failure also represents the most frequent cause of hospitalization, and about 30% of the patients will undergo re-hospitalization within 60 to 90 days after being discharged from the hospital [4].

Digoxin is the oldest drug used for the treatment of heart failure. Despite questionable long-term efficacy and safety, and the fact that digoxin has been less prescribed anno 2022, however, in some (developing) countries, including our country, many physicians still prescribe digoxin, especially for treating heart failure with atrial fibrillation, tachycardia and/or low ejection fraction, because digoxin is an effective drug for controlling heart rate and for increasing ejection fraction. Thus, this drug is continuously being used until nowadays [5]. Study conducted by Ziff *et al.* reported that digoxin can significantly improve clinical symptoms and reduce hospitalization, but not mortality [6]. Digoxin has a narrow margin of safety: Dose reduction may lead to inefficacy, while dose increment may lead to adverse effects, or even, intoxication. Local data from the National Cardiovascular Center in Jakarta from 2017-2018 reported 225 measurements of plasma digoxin level. From those patients, 148 (65%) showed a supra-therapeutic plasma level. According to the literature, plasma levels above 2 ng/mL may lead to intoxication with possible manifestation as arrhythmia (90%), gastrointestinal symptoms (55%), and neurological symptoms (12%) [7]. Severe intoxication may lead to death, with the incidence between 20-30% [8].

In Indonesia, digoxin is still used as one of the treatments for heart failure. However, analyses on intoxication have never been reported. This study aimed to analyze and report the occurrence of digoxin intoxication in heart failure patients at Harapan Kita National Heart Center, Jakarta, Indonesia.

## 2 MATERIALS AND METHODS

This was an observational analytic study with cross-sectional design conducted at Harapan Kita National Cardiovascular Centre, Jakarta, Indonesia. Digoxin intoxication was defined according to the presence of specific ECG abnormality; extra-cardiac symptom(s) including gastrointestinal, neurological and/or ophthalmologic symptom(s); and further classified as definite, probable, or possible if serum digoxin level is respectively toxic (≥ 2.0 ng/mL), supra-therapeutic (0.91-1.99 ng/mL), or therapeutic (0.5 - 0.9 ng/mL) [9]. Whereas, those with sub-therapeutic digoxin levels (0.06 - 0.49 ng/mL) are classified as no intoxication.

Some risk factors for digoxin intoxication were also analyzed, including age (< 65 or > 65 years old), gender, renal insufficiency (eGFR < 60 ml/min/1.73 m^2^, or creatinine level > 2.5 mg/dL), electrolyte imbalance including hypokalemia (K < 3.3 mEq/L), hyperkalemia (K > 5.5 mEq/L), hypcalsemia (Ca <8.8 mEq/L), hypercalsemia (Ca > 10.4 mEq/L), hypomagnesema (Mg <1,8 mEq/L), hypermagnesimia (Mg > 2.2 mEq/L), and drug-drug interaction was defined as the presence of coadministration of digoxin with the drug of concern which were furosemide, tiazides, spironolactone, ACE-inhibitor, ARB, beta blocker, amiodaron, verapamil, diltiazem, rifampisin, and aminoglycoside.

Mortality and rehospitalization were recorded for one year of digoxin treatment. All data were taken from the medical records of the hospital. Sample size of this study was based on total patients with heart failure that received digoxin treatment and had at least one measurement of plasma digoxin level between the period of January 2017 to December 2018. In the case of several measurements, only the first one was taken for analysis of intoxication. Measurement of digoxin levels was performed by the hospital laboratory which uses KIM (Kinetic Interation of Microparticle in Solution) methods by using commercial Kit from Roche. Neonates, infants, and patients with incomplete medical record data were excluded.

Categorical data of intoxication was analyzed by Chi-squared test, while mortality and rehospitalization were analyzed with Kaplan Meyer test. The protocol of this study has been approved by the Institutional Review Board of Harapan Kita National Heart Centre with approval letter: LB.01.02/XX.8/1053/2019.

## 3 RESULTS

Baseline characteristics of patients can be seen in Table **1**. A total of 195 have been included in this study, consisting of 114 females and 81 males with a mean age of 51 ± 17 years (ranging 11 - 91 years). Subtherapeutic digoxin levels were observed in 22 patients (11.28%); therapeutic level in 43 patients (22.05%); supratherapeutic level in 70 patients (35.90%); and toxic level in 60 patients (30.77%).

According to the presence of typical ECG abnormality, extra-cardiac symptom(s), and serum digoxin level, the occurrence of digoxin intoxications were found as follows: 32 patients (16,4%) definite, 19 patients (9.74%) probable, and 3 patients (1.54%) possible intoxication (Table **2**).

ECG alterations in study subjects are shown in Table **3**.

Analysis of risk factors can be seen in Table **4**.

From Table **4**, it can be seen that the only significant risk factor for digoxin intoxication is renal insufficiency.

Survival analysis is depicted in Fig. (**1**).

Mean survival of those with- and without intoxication are respectively 259 and 307 days (*p =* 0.063) days with overall survival of 300 days.

### 3.1 Rehospitalization

Thirty-four of 195 patients died during hospitalization. Thus, analysis of rehospitalization is only conducted on 161 patients and was followed-up for one year. Digoxin administration was continued in those with normal ECG, and discontinued in those with abnormal ECG. Median time to rehospitalization was 49 days (CI 95%: 36-62) in those receiving digoxin, and 14 days (CI 95%: 3-25) in those without digoxin.

Proportions of rehospitalization in group receiving and not receiving digoxin were 11.8% and 29.2%, respectively (*p =* 0.085) (Table **5**).

## 4 DISCUSSION

Digoxin is one of the oldest drugs used for the treatment of heart failure [10-12]. Although its use in acute conditions significantly improves heart failure symptoms, long-term use failed to show a decrease in mortality. The availability of newer drugs with better safety profiles such as ACE-inhibitor/ARB/ARNI, beta blocker, and MRA and its effects in decreasing mortality, has led to lesser use of digoxin in the last two decades [13]. However, nowadays, some physicians continue to use digoxin for reasons such as its efficacy, low cost, wide availability, and the feeling that they have sufficient experience with this drug and its good safety profile with careful dose titration [14]. The report of Digitalis Investigation Group (DIG) showed that digoxin significantly reduces the number of hospitalizations of heart failure, reduces the hospital length of stay, and improves functional class of heart failure, although not reducing mortality [15]. Indications of digoxin use have shifted from the main treatment of heart failure in the past, to supraventricular tachycardia, and finally as an alternative treatment of atrial fibrillation, especially if accompanied by heart failure. The effects of digoxin in atrial fibrillation are mediated by its vagotonic effect, thus reducing heart rate, and prolongation of refractory period in AV node, hence preventing the transmission of fibrillation from the atrium to the ventricle [16]. However, in toxic doses, excessive suppression of AV nodes may result in AV block, and the increased automaticity on ventricular muscle may lead to ventricular arrhythmia, and even fibrillation [7, 10].

In the present study, digoxin intoxication was diagnosed when the patient showed typical ECG changes and at least one extra-cardiac symptom(s) and classified as “definite”, when serum digoxin level falls to toxic level (> 2 ng/mL), “probable”, if serum digoxin level is supratherapeutic (0.91-1.99 ng/mL), and “possible”, if serum digoxin level falls in therapeutic range (0.5-0.9 ng/mL). We took into account the probable and possible intoxication, although serum digoxin level did not reach toxic levels since some individuals may have different sensitivity to toxic symptoms.

Although the dose of digoxin administered to the patients was intended to obtain optimal serum therapeutic level, this study showed that about two third of patients have their digoxin level above therapeutic range (supra-therapeutic or toxic levels), and one third have therapeutic or subtherapeutic levels. These differences can be explained by several possibilities. First, adult patients are normally given the same dose without considering variation of body weight. Thus, those with low body weight will have higher serum levels compared to those with normal or high body weight. Another study with standardized digoxin dose (10 ug/kg/d) and timing of blood sampling just before digoxin administration is reported by Nafrialdi *et al*. in the Department of Child Health, Dr. Cipto Mangunkusumo Hospital, Jakarta [18]. It was found that 13 out of 20 patients (65%) had plasma digoxin levels within therapeutic range (0.5-1.5 ng/mL; 95%CI 0.599 to 0.898) and 7 (35%) had sub-therapeutic levels (<0.5 ng/mL; 95%CI 0.252 to 0.417), and no case of supratherapeutic level was found.

Second, drug absorption may be influenced by the presence of food or other drugs at the time of drug ingestion. As noted, bioavailability of digoxin may vary up to 40% when taken in empty or full stomach. Third, the time of blood sampling may have a significant influence on serum digoxin level. In our study population, the timing of blood sampling is not rigorously controlled. In daily practice, the physicians will order measurement of drug level in case of suspicions of drug intoxication, thus the timing of blood sampling may be varied. In fact, the best time for blood sampling is between 6-8 hours after ingestion for allowing stabilization of blood and tissue distribution. If blood sampling is taken too early, higher serum levels would be obtained while distribution to cardiac tissue is still in process. Whereas cardiac manifestation as the primary indicator of digoxin intoxication is mainly governed by drug level in cardiac tissue. Beyond all above factors, sensitivity to toxic manifestation may be different from one to others, although serum digoxin level may be the same.

In the present study, the most frequent cardiac manifestations of digoxin intoxication were typical ECG alterations (46.7%), while extracardiac manifestations were mostly detected as gastrointestinal symptoms (49.7%), followed respectively by neurologic and ophthalmic symptoms. These findings are in line with previous studies which showed that GI symptoms such as nausea and vomitus, were the most frequent manifestation (30-80%) especially in female patients; followed by neurologic manifestation in the forms of dizziness, malaise, weakness, and ophthalmic symptoms in the form of green-yellow colour blindness [11, 14].

ECG alterations compatible with digoxin intoxication consisted of ventricular extrasystole (VES)/ premature ventricular contraction (PVC), bigeminy, grade I-III AV block, total AV-block (TAVB), accelerated junctional, AF-SVR, RBBB/LBBB, VT-bidirectional. Combination of augmented automaticity and conduction disturbances such as AV block and accelerated junction, indicates great probability of intoxication, although digoxin serum level is in therapeutic range [11]. In that situation, the physicians normally send the blood specimen for measurement of serum digoxin level. It is revealed that 67% of patients have high digoxin serum levels, consisting of 36% supratherapeutic level (0.91-1.99 ng/mL) and 31% toxic level (> 2 ng/mL). However, clinical manifestation and specific ECG alteration do not necessarily occur.

Digoxin is a drug well known for its narrow margin of safety [7, 10]. The increment of dose may lead to adverse effects ranging from subjective symptoms in the form of nausea, vomitus, blurred vision, neurologic symptoms, and various forms of arrhythmias until ventricular fibrillation. Certain comorbidities such as electrolyte imbalance, renal dysfunction, and older age are associated with a higher risk of intoxication [8, 11, 12].

Beller *et al.* [17] reported in 1970, that 25% of heart failure patients treated with digoxin were definitively diagnosed as having intoxication, while 6% only showed symptoms and signs. However, in recent years, the incidence of digoxin intoxication has significantly decreased due to the reduced use of this drug [18]. Between 1980-1990, the incident of digoxin intoxication was reported around 4-5% [19]. In 2005-2010, See *et al.* reported that in the US around 1% of patients receiving digoxin showed symptoms of digoxin intoxication; the incidence of intoxication increased along with the increase of age in the elderly. Thus, monitoring of plasma digoxin level is mandatory in this special age group [20].

In the present study, there were 31.2% (10/32) of patient deaths associated with intoxication. There was a 1.9-fold increased risk of mortality in patients with intoxication compared to those without intoxication, although the difference was not clinically significant (*p=*0.063). Variation in the severity of background illness might contribute to mortality.

Renal insufficiency was proven as a significant risk factor for digoxin intoxication. In the present study, 59% of patients had renal insufficiency, and this factor was associated with a 2.5-fold increase in intoxication (*p =* 0.016, RR 2.484, CI 95% 1.130-5.464), and the duration of intoxication was also longer.

Concerning rehospitalization, treatment with digoxin is associated with a lower incidence of rehospitalization (11.8% vs 29.2%) and longer interval to rehospitalization (49 *vs* 14 days), although these differences were statistically non-significant (*p=*0.085). These findings are in line with the study by Ahmed *et al*. [21] in the US which reported lower incidence of rehospitalization due to many causes in patients treated with digoxin after 30 days and 1 year of observation. The PROVED trial evaluated the effect of digoxin withdrawal on heart failure patients previously treated with digoxin and diuretic. It was proven that the patients in the withdrawal group showed significant worsening of exercise capacity (*p=*0.003), lower ejection fraction (*p=* 0.016), and increased incidence of heart failure exacerbations (*p=*0.039) [22]. In RADIANCE trial, 178 heart failure patients with ejection fraction of < 35% and NewYork Heart Association (NYHA) class II–III symptoms on a stable regimen of digoxin, diuretics, and an ACE-inhibitor (captopril or enalapril) were randomized to withdrawal or continuation of digoxin for 12 weeks. There was a significantly higher rate of worsening of heart failure, with a relative risk of 5.9 (95% CI 2.1–17.2) in the withdrawal group compared to the digoxin group [23].

### 4.1 Strength and Limitation of the Study

Although the problem of digoxin intoxication is considered an old issue, this drug is still used in our country, and the report on this problem is scarce. This study is the first to report the data on digoxin intoxication at Harapan Kita Hospital, which is the biggest Cardiovascular center in our country. Additionally, according to our knowledge, this is the first to classify dogoxin intoxication into definite, probable, and possible. This classification was made based on the fact that different individuals may have different sensitivity to the risk of intoxication.

On the other side, this study also has same limitation, including non-standardize timing of blood sampling for measurement of digoxin levels and the fact that the dose of digoxin was not specifically adjusted according to body weight. In addition, the retrospective nature of this study also contributed to its limitation of this study.

## CONCLUSION

From the above study, it can be concluded that the proportion of digoxin intoxication is 27.69%. Renal insufficiency was proven as a significant risk factor. There was a tendency of lower incidence and longer interval to rehospitalization in patients receiving digoxin.

## Figures and Tables

**Fig. (1):**
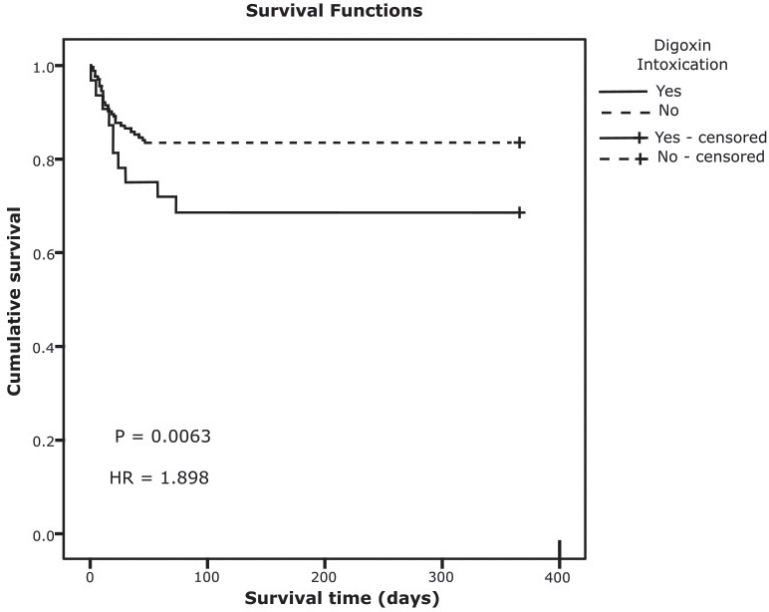
One year survival of patients with- and without digoxin intoxication.

**Table 1:** Baseline characteristic of study subjects (*n=* 195).

**Variable**	**n (%)**
Age (year, *mean ± SD*, *range*)	51 ± 17 (11-91)
Gender:	
Male	81 (41.5)
Female	114 (58.5)
Median serum digoxin level (ng/mL)	1.38 (0.06-11.4)
Category of digoxin level	
Sub-therapeutic	22 (11.3)
Therapeutic	43 (22.1)
Supra-therapeutic	70 (35.9)
Toxic	60 (30.8)
Indication of digoxin:	
Heart failure	43 (22.1)
Heart failure + Atrial fibrillation	152 (77.9)
Cause of HF	
Valvular HD*	110 (56.4)
Ischemic HD	63 (32.3)
Congenital HD	19 (9.7)
Hypertensive HD	3 (1.5)
Mortality, n (%):	
Survive	158 (81)
Dead	37 (19)
Rehospitalization, n (%)	
With digoxin	21 (10.8)
Without digoxin	46 (23.6)
Complications, n (%)	
Kidney disease	115 (59)
Electrolyte imbalance	134 (68.7)
Drug interaction	159 (81.5)
Specific ECG abnormality	91 (46.7)
Extra-cardiac symptoms, n (%)	
GI: nausea, vomiting, diaren, abdominal pain	97 (4.7)
Neurologic: seizure, headache, lethargic	39 (20)
Ophthalmologic: blurred vision	8 (4.1)

**Note:** *Valvular heart disease: mitral, tricuspid, aortic, pulmonary.

GI: gastrointestinal.

HD: heart disease.

**Table 2:** Classification of intoxication in study subjects.

**Classification of Intoxication**	**N**	**%**
Definite	32	16.41
Probable	19	9.74
Possible	3	1.54
No intoxication	141	72.31
Total	195	100

**Table 3:** ECG change(s) in patients receiving digoxin.

-	**n (%)**
**Non-typical ECG Change**	**110 (53.3)**
**ECG Change(s) Suspecting Digoxin Intoxication:**	**96 (46.7)**
First-degree, second-degree (Wenckebach), or third-degree heart block (including TAVB, asystole)	8 (4.1)
LBBB or RBBB	24 (12.3)
Atrial tachycardia with block, atrioventricular dissociation	4 (2.1)
Accelerated junctional (nodal) rhythm	3 (1.5)
Premature ventricular contractions (PVC), especially bigeminy or trigeminy	10 (5.1)
Ventricular tachycardia and ventricular fibrillation, bidirectional	2 (1.0)
Atrial fibrillation or atrial flutter, slow ventricular response.	25 (12.8)
Ventricular ExtraSystole (VES), bigeminy	13 (6.7)
AV nodal reentrant tachycardia (AVNRT),	1 (0.5)
Torsade de pointes	1 (0.5)

**Table 4:** Risk factors of digoxin intoxication.

**Variable**	**Intoxication**		**RR**	**CI95%**	**p**
	**Yes, n (%)**	**No, n (%)**	-	-	-
Age	-	-	-	-	-
< 65 years	25 (12.8)	129 (66.2)	0.951	0.443-2.041	0.897
> 65 years	7 (3.6)	34 (17.4)	-	-	-
Gender	-	-	-	-	-
Male	13 (6.7)	68 (34.9)	0.963	0.505-1.836	0.909
Female	19 (9.7)	95 (48.7)	-	-	-
Renal insufficiency*	-	-	-	-	-
Yes	25 (12.8)	90 (46.2)	2.484	1.130-5.464	0.016
No	7 (3.6)	73 (37.4)	-	-	-
Electrolyte imbalance^$^	-	-	-	-	-
Yes	25(12.8)	109(55.9)	1.626	0.744-3.552	0.209
No	7(3.6)	54(27.7)	-	-	-
Drug interaction#	-	-	-	-	-
Yes	27 (13.8)	132 (67.7)	1.223	0.506-2.957	0.651
No	5 (2.6)	31 (15.9)	-	-	-

**Note:** * eGFR < 60 ml/min./1.73 m^2^, or creatinine level > 2.5 mg/dL in case of the absence of information of body weight or hight.

^$^hypokalemia, hyperkalemia, hypocalsemia, hypercalsemia, hypomagnesemia, hypermagnesemia. (Normal ranges are stated in the Methods)

# coadministration of digoxin with either one of the following drugs: furosemide, thiazide, spironolactone, ACE-inhibitors, ARB, amiodaron, verapamil, diltiazem, rifampicin, aminoglycoside.

**Table 5:** Proportion of rehospitalization.

**Variable**	**Rehospitalization**		***P***	**RR**	**CI95%**
	**Yes**	**No**			
Drug	-	-	-	-	-
Digoxin (+)	19 (11.8)	40 (24.8)	0.085	0.699	0.457-1.070
Digoxin (-)	47 (29.2)	55 (34.2)	-	-	-

## Data Availability

The data supporting the findings of this study are available within the article.
